# Use of combined maximum and minimum intensity projections to determine internal target volume in 4-dimensional CT scans for hepatic malignancies

**DOI:** 10.1186/1748-717X-7-11

**Published:** 2012-01-30

**Authors:** Jin Liu, Jia-Zhou Wang, Jian-Dong Zhao, Zhi-Yong Xu, Guo-Liang Jiang

**Affiliations:** 1 Department of Radiation Oncology, Fudan University Shanghai Cancer Center. 270 Dong An Road, Shanghai, 200032, China

**Keywords:** liver malignancy, radiotherapy, internal target volume, 4-dimensional CT, maximum intensity projection, minimum intensity projection

## Abstract

**Background:**

To evaluate the accuracy of the combined maximum and minimum intensity projection-based internal target volume (ITV) delineation in 4-dimensional (4D) CT scans for liver malignancies.

**Methods:**

4D CT with synchronized IV contrast data were acquired from 15 liver cancer patients (4 hepatocellular carcinomas; 11 hepatic metastases). We used five approaches to determine ITVs: (1). ITV_AllPhases_: contouring gross tumor volume (GTV) on each of 10 respiratory phases of 4D CT data set and combining these GTVs; (2). ITV_2Phase_: contouring GTV on CT of the peak inhale phase (0% phase) and the peak exhale phase (50%) and then combining the two; (3). ITV_MIP_: contouring GTV on MIP with modifications based on physician's visual verification of contours in each respiratory phase; (4). ITV_MinIP_: contouring GTV on MinIP with modification by physician; (5). ITV_2M_: combining ITV_MIP _and ITV_MinIP_. ITV_AllPhases _was taken as the reference ITV, and the metrics used for comparison were: matching index (MI), under- and over-estimated volume (V_under _and V_over_).

**Results:**

4D CT images were successfully acquired from 15 patients and tumor margins were clearly discernable in all patients. There were 9 cases of low density and 6, mixed on CT images. After comparisons of metrics, the tool of ITV_2M _was the most appropriate to contour ITV for liver malignancies with the highest MI of 0.93 ± 0.04 and the lowest proportion of V_under _(0.07 ± 0.04). Moreover, tumor volume, target motion three-dimensionally and ratio of tumor vertical diameter over tumor motion magnitude in cranio-caudal direction did not significantly influence the values of MI and proportion of V_under_.

**Conclusion:**

The tool of ITV_2M _is recommended as a reliable method for generating ITVs from 4D CT data sets in liver cancer.

## Introduction

Primary and metastatic hepatic malignancies are commonly treated by surgery, but radiation therapy is also one of options as non-surgical modalities. It has been demonstrated that radiation therapy is feasible and the outcomes are promising [1,2]. However, due to respiration liver motion up to 3 cm [3] is one of obstacles to accurately localize the target. Moreover, respiratory-induced tumor motion is known to be anisotropic, thus individual determination of internal margin around gross tumor volume (GTV) is crucial to form an internal target volume (ITV), which can avoid both inadequate tumor coverage and unnecessary liver parenchymal irradiation for individual patient. Four-dimensional computed tomography (4D CT) is one of appropriate approaches to estimate and determine ITV for tumor with respiratory motion [4,5].

4D CT has been widely used in lung cancer to determine ITV [6,7]. Ideally, ITV should be delineated by manually contouring GTV in all 10 breath phases of a 4D scan image sets to form ITV, which is the most accurate tool to determine ITV, but it is a time-consuming and labor-intensive task. To reduce the workload of contouring multiple GTVs, one solution is to contour only two extreme phases at end-inhalation and end-exhalation and then to sum of the two becoming ITV [8,9]; and the other is to use the post-processing tools of maximum intensity projection (MIP) and minimum intensity projection (MinIP) from 4D CT data sets to generate ITV. MIP-based ITV delineation is performed on a single 3-D CT data set, where each pixel in this set represents the brightest object encountered by corresponding voxels in all volumetric 4D CT data sets, for instance, MIP-based ITV delineation for lung cancer, which was recommended as a reliable tool and a good first estimation [10-12]. Conversely, MinIP-based ITV is on the CT set, where each pixel represents the lowest data value in the volumetric data [10].

MIP and MinIP methods seem not suitable for liver cancer because most tumors in the liver have similar attenuation to the normal liver parenchyma and therefore are not easily discernable. Contrast-enhanced CT scan has been routinely used for radiation oncologists to differentiate the tumor from normal tissues. It should be noted that regardless of with contrast enhancement or not, most liver tumors present inhomogeneous density, either because of the inherent nature of the tumor, such as the routes of blood supply, vascular volume and permeability, or because of areas of fluid, hemorrhage, and necrosis within tumors, or because of secondary change due to treatments, for example, iodine deposition as a result of transcatheter arterial chemoembolization (TACE). Recently, Beddar et al described a simple method for 4D CT acquisition by using synchronized intravenous contrast injection to improve the accuracy of liver tumor delineation. By this way most liver metastases and cholangiocarcinomas can be identified on image of portal venous phase, while HCC, most visible in the delayed phase [13].

In theory, for tumors with homogeneous hyperdensity or hypodensity comparing to the surrounding normal liver, MIP or MinIP projections should accordingly reflect the tumor trajectory across all time-resolved data sets. Visualization of tumor with mixed-density means the tumor border should be discernable; regardless which part of it is more hyperdense or hypodense than the adjacent liver parenchyma. Using MIP technique or MinIP technique only definitely misses the spatial information of the moving liver tumor. Therefore, our hypothesis is to combine MIP and MinIP, which may fit for the situation of mixed tumor density. Thus, the purpose of this study is to evaluate the feasibility and accuracy of the MIP and/or MinIP-based ITV delineation in 4DCT scans for liver tumors.

## Methods

### Patient selection

Patients who met the following criteria were qualified to this study: (1). Patient suffered from hepatic malignancies, primary or metastatic, and were planned to receive irradiation; (2). The margins of hepatic lesion were clear on intravenous contrast enhanced CT; (3). Patient's breath was regular after a training session; (4). Patient did not have the history of allege to contrast; and (5). Informed consent was obtained.

### 4D CT image acquisition

In order to enhance the visibility of tumors on 4D CT, 100 mL of contrast at a concentration of 300 mg I/mL was injected synchronously with 4D CT image acquisition. All patients were imaged during the portal venous phase. A time delay was programmed within 4D CT image acquisition protocol so that the start of contrast injection is initiated simultaneously with the start of the scanner's timer countdown. For those with liver metastases, the liver was scanned with a 45 s time delay, while for hepatocellular carcinoma (HCC) patients the time delay was 65 s. This CT scan protocol was proposed by Beddar et al [13].

All patients were immobilized using customized vacuum lock in supine position with arms placed on their forehead. A16-slice Brilliance Big Bore CT scanner (Philips Co.) was used for 4D CT image acquisition. The patient respiration was tracked using Real-Time Position Management (RPM) System (Varian Medical Systems, Palo Alto, CA). The region of interest usually comprises the area from 2 to 3 cm above diaphragm to iliac crest. A 4D CT scan is performed in cine mode with at least one complete breath cycle for each couch position. After scanning 4-dimensional images were binned based on the respiratory traces to become a complete image set, which covered each of 10 breathing phases. MIP and minimum intensity projections (MinIP) were then generated from the raw data set of 4D CT scans.

### ITV determination

**Figure 1:**
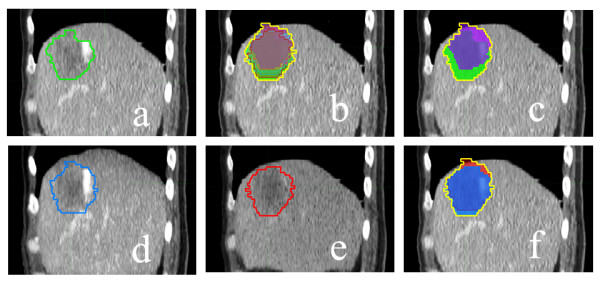
**Panel (a) shows the GTV (green contour) for patient 13; Delineation of ITV base on ITV_AllPhases_, ITV_2Phase_, ITV_MIP_, ITV_MinIP _and ITV_2M _are shown in panels (b), (c), (d), (e) and (f), respectively**. ITV_MIP _and ITV_MinIP _coutours are as they appear on the intensity projection data set; all others are registered to the 0% phase of the 4D CT data set.

All reconstructed CT series were transferred to MIM software (Version 5.1, MIMvista Corp., Cleveland, OH). Window/level was adjusted to optimize the visual contrast between the tumor and normal parenchyma regions. All the contours were drawn by a single radiation oncologist (JL) and verified by a senior radiation oncologist (JDZ). We used 5 approaches to determine ITV, which were: (1). ITV_AllPhases_: contouring GTV on each of 10 respiratory phases of 4D CT data set and combining these GTVs; (2). ITV_2Phase_: contouring GTV on CT of the peak inhale phase (0% phase) and the peak exhale phases (50%) and then combining the two; (3). ITV_MIP_: contouring GTV on MIP with modifications based on physician's visual verification of contours in each respiratory phase; (4). ITV_MinIP_: contouring GTV on MinIP with modification by physician; (5). ITV_2M_: combining ITV_MIP _and ITV_MinIP_. Figure 1 illustrates different approaches in the determination of ITV for patient 13.

### Evaluation of target motion

We used MIM software (version 5.1) to measure cranio-caudal, left-right and anterior-posterior movement of the tumor. Tumor motion was also expressed as a 3D vector, which is the quadratic mean of the motions in 3 orthogonal directions.

### Data analysis and statistics

ITV_2Phase_, ITV_MIP_, ITV_MinIP _and ITV_2M_, as the tested ITVs, would be compared with the reference of ITV_AllPhases_, respectively. The metrics used for comparison were: (1) Matching index (MI), which was the percentage of overlapped volume in 3-dimensions between 2 ITVs. When 2 ITVs were totally overlapped, MI was 1.00, whereas when 2 ITVs were totally separate, MI was 0; and (2) under- and over-estimated volume (V_under _and V_over_). A tested ITV (V _test_) was compared to the standard volume, ITV_AllPhases_. The formulas were: V_under _*= *V_AllPhases_\V_test_, and V_over _*= *V_test _\ V_AllPhases_.

VUnder=∫Aallphase(Z)\Atest(Z)dz

VOver=∫Atest(Z)\Aallphase(Z)dz

The definitions and calculations of those metrics were referred to Ezhil [12].

Paired sample *t*-test and Independent-Samples T test (SPSS v.13, SPSS Inc., Chicago, IL) were applied to compare the differences with *p *value of < 0.05 considered significant.

## Results

### Patients

From August 2010 to February 2011, 15 eligible patients with liver cancer were enrolled in this study and underwent 4D CT simulation for irradiation treatment planning in our institution. Of these patients 11 had metastatic liver cancers and 4, HCC with mean lesion volume of 152 cm^3 ^(range, 2 cm^3 ^- 932 cm^3^). 4D CT images were successfully obtained from 15 patients and tumor margins were clearly discernable in all patients. There were 9 cases of low density and 6, mixed on CT images (Table 1).

**Table 1:** Tumor Characteristics

Patient	Tumor	Tumor size* (mm)	GTV(cm^3^)	Location	Tumor density
1	HCC	51 × 42 × 23	34	Right upper lobe; adjacent to right lung.	Mixed
2	LM from lung cancer	33 × 49 × 33	30	Left lobe; intrahepatic	Low
3	LM from gastric carcinoma	42 × 78 × 52	77	Caudate and left lobe; intrahepatic	Low
4	LM from pancreatic carcinoma	42 × 51 × 48	66	Right lower lobe; intrahepatic	Mixed
5	LM from nasopharyngeal carcinoma	12 × 15 × 15	2	Right upper lobe; intrahepatic	Low
6	LM from rectal carcinoma	10 × 12 × 11	2	Right upper lobe; intrahepatic	Low
7	HCC	35 × 39 × 48	59	Right upper lobe; adjacent to right lung	Mixed
8	LM from gallbladder carcinoma	9 × 18 × 18	3	Right lower lobe; intrahepatic	Low
9	LM from gastric carcinoma	51 × 99 × 58	213	Left lobe; adjacent to stomach	Low
10	HCC	78 × 96 × 99	384	Right lobe; adjacent to right lung and kidney	Mixed
11	LM from lung cancer	50 × 59 × 45	80	Left lobe; adjacent to stomach	Low
12	LM from gallbladder carcinoma	33 × 38 × 42	28	Right lobe; intrahepatic	Low
13	LM from rectal carcinoma	54 × 56 × 54	91	Right upper lobe; adjacent to right lung	Mixed
14	HCC	108 × 126 × 145	932	Right upper lobe; adjacent to right lung	Mixed
15	LM from esophageal carcinoma	60 × 91 × 88	284	Right lobe; adjacent to right lung and kidney	Low

*Abbreviations: *GTV: gross tumor volume; HCC: hepatocellular carcinoma; LM: liver metastasis.

*Tumor size was expressed as multiplying diameters in cranio-caudal, in left-right and in anterior-posterior directions.

### Target motion

The cranio-caudal motion of the target was predominant with a mean distance of 8.0 mm ± 3.3 mm, while the left-right and anterior-posterior motions were much less with mean values of 1.6 mm ± 0.9 mm and 3.2 mm ± 2.2 mm, respectively (Table 2).

**Table 2:** Motion magnitudes of GTV centroid measured by 4D CT in 15 patients (mm)

Patient	Cranio-caudal	Left-right	Anterior-posterior	3D vector
1	8.8	2.9	2.3	9.6
2	11.9	1.1	1.4	12.0
3	6.9	1.0	2.8	7.5
4	8.8	1.3	3.0	9.4
5	8.7	1.7	2.2	9.1
6	10.0	1.1	3.9	10.8
7	15.2	2.5	8.1	17.4
8	7.6	3.6	4.3	9.5
9	8.0	1.1	1.4	8.2
10	5.3	2.7	8.2	10.1
11	3.4	1.0	3.2	4.8
12	3.0	1.0	2.1	3.8
13	11.5	1.7	2.3	11.9
14	3.9	0.9	0.7	4.1
15	7.4	1.0	1.7	7.7
Mean	8.0	1.6	3.2	9.1
SD	3.3	0.9	2.2	3.4

### Comparison of ITVs countered by different approaches

#### (1). ITVs volume

Table 3 shows all volumes of ITV. The mean volume of ITV_2M _was closest to that of ITV_AllPhases_, and then followed by ITV_2Phase_. The volume difference between ITV_2M _and ITV_2Phase _was statistically significant with a *p *value of 0.04. Taking ITV_AllPhases _as the reference, the mean ratios of the tested ITVs to ITV_AllPhases _were 88.9% ± 5.7%, 82.7% ± 12.6%, 82.5% ± 10.8% and 94.0% ± 3.6%, respectively for ITV_2phase_, ITV_MinIP_, ITV_MIP _and ITV_2M_.

**Table 3:** Volumes of ITV_AllPhases_, ITV_2M_, ITV_2Phase_, ITV_MinIP _and ITV_MIP _(cm^3^)

Patient	ITV_AllPhases_	ITV_2M_	ITV_2Phase_	ITV_MinIP_	ITV_MIP_
1	52.8	44.2	43.2	32.5	41.5
2	50.9	48.8	47.1	43.7	43.6
3	112.6	101.4	102.4	97.5	80.9
4	100.1	94.8	91.2	70.9	94.5
5	5.7	5.1	4.3	5.0	4.1
6	5.0	4.7	4.1	4.6	2.9
7	96.1	91.4	85.3	56.0	90.0
8	6.8	6.5	5.9	6.5	5.0
9	294.8	283.0	280.6	233.3	275.2
10	569.3	549.6	514.6	390.3	528.7
11	99.5	95.7	88.9	93.8	86.2
12	40.3	38.9	37.2	38.8	30.1
13	123.2	116.8	110.0	98.9	104.9
14	1082.7	1041.1	1040.3	963.8	1013.6
15	372.0	357.6	347.3	355.7	312.5
Mean	200.8	192.0	186.8	166.1	180.9
SD	290.4	280.0	277.0	252.0	272.0

#### (2). MI

As shown in Table 4, ITV_2M _was closest matched with ITV_AllPhases _with mean MI of 0.93 ± 0.04, and mean MIs were 0.89 ± 0.06, 0.82 ± 0.12 and 0.82 ± 0.10, respectively for ITV_2Phase_, ITV_MinIP _and ITV_MIP_. The differences of MI were statistically significant between ITV_2M _and ITV_2Phase _(*p *= 0.004), between ITV_2M _and ITV_MinIP _(*p *= 0.003), and between ITV_2M _and ITV_MIP _(*p *= 0.000). All the other comparisons between ITVs were not significant.

**Table 4:** MI values for four ITVs based on ITV_2M_, ITV_2Phase_, ITV_MinIP _and ITV_MIP _relative to the reference ITV_AllPhases_

Patient	ITV_2M_	ITV_2Phase_	ITV_MinIP_	ITV_MIP_
1	.83	.82	.61	.78
2	.94	.92	.86	.83
3	.89	.91	.86	.72
4	.93	.91	.71	.93
5	.89	.75	.87	.73
6	.91	.81	.91	.58
7	.94	.89	.58	.93
8	.95	.87	.95	.72
9	.94	.95	.79	.92
10	.95	.90	.68	.92
11	.95	.89	.94	.86
12	.95	.92	.95	.75
13	.93	.89	.80	.84
14	.96	.96	.89	.93
15	.96	.94	.95	.84
Mean	.93	.89	.82	.82
SD	.04	.06	.12	.10

We further analyze the tumor characteristics, which would impact MI.

**Figure 2:**
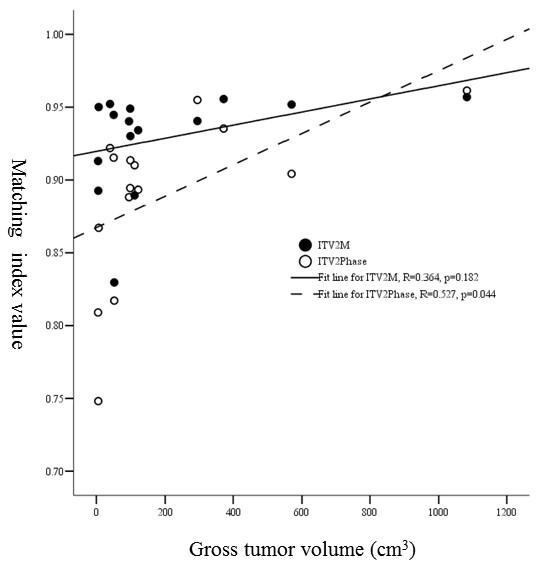
**Relationship between gross tumor volume and MI values of ITV_2M _and ITV_2Phase_**.

**1). MI and tumor volume: **As lesion of > 5 cm is not a candidate for stereotactic body radiation therapy in our practice, the patients were split into two groups: ≤ 65.4 cm^3 ^(equivalent to the volume of a sphere with 5 cm in diameter) vs. > 65.4 cm^3^. MIs of ITV_2M _were 0.94 ± 0.02 for the former and 0.92 ± 0.04 for the latter (*p *= 0.260). Nevertheless, there was significant difference between tumor size and MIs of ITV_2Phase_, MI being 0.92 ± 0.03 for tumor of ≤ 65.4 cm^3 ^and 0.85 ± 0.06 for tumor of > 65.4 cm^3 ^(*p *= 0.015). For ITV_2M _as tumor volume increased MI did not change much with no correlation between them (R = 0.364, *p *= 0.182). However, for ITV_2Phase_, when tumor volume increased MI was significantly enhanced with positive correlation (R = 0.527, *p *= 0.044) (Figure 2).

**2). MI and target motion three-dimensionally: **It is recommended that for tumor motion of > 10 mm, we need to reduce the movement by breath control devices, such as Active Breathing Coordinator or RPM gating system. The patients were divided into two groups: 3D vector of ≤ 10 mm vs. > 10 mm. Mean MIs of ITV_2M _were 0.94 ± 0.01 and 0.92 ± 0.04 (*p *= 0.542), and mean of MIs of ITV_2Phase_, 0.88 ± 0.04 and 0.89 ± 0.06 (*p *= 0.756), respectively for the former and the latter. There was no significant correlation between the magnitude of target motion and MI.

**Figure 3:**
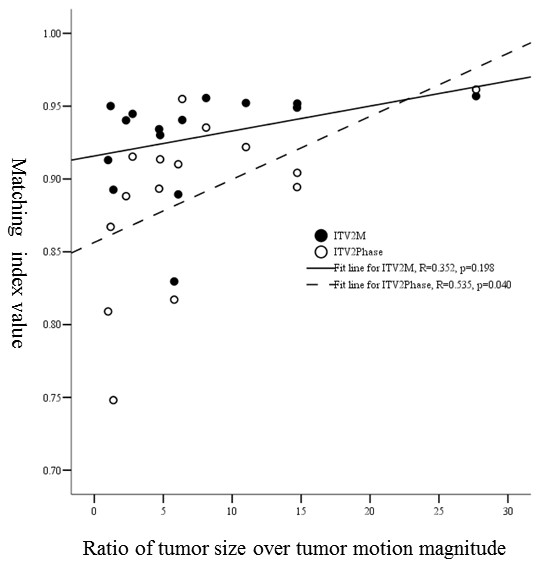
**The relationship between the ratio of tumor vertical diameter over tumor motion magnitude in cranio-caudal direction and MI values of ITV_2M _and ITV_2Phase_**.

**3). MI and ratio of tumor vertical diameter over tumor motion magnitude in cranio-caudal direction: **As shown in Figure 3, for ITV_2M _when vertical diameter over tumor motion magnitude in cranio-caudal direction increased MI was increased slightly, but with no significant correlation (R = 0.352, *p *= 0.198). However, for ITV_2Phase _there was a positive correlation between them (R = 0.535, *p *= 0.040).

**4). MI and tumor density: **For the tumor of low density or mixed density, there was no significant difference in MIs between them for both ITV_2M _and ITV_2Phase_, MIs of ITV_2M _being 0.93 ± 0.02 and 0.92 ± 0.05 (*p *= 0.676), and MIs of ITV_2Phase_, 0.88 ± 0.07 and 0.90 ± 0.05 (*p *= 0.702), respectively for low density tumor and the mixed. However, for low density tumor MI of ITV_MinIP _was better than that for mixed density tumor with MI of 0.90 ± 0.05 and 0.71 ± 0.11 (*p *= 0.001), respectively.

We also noticed that for low density tumors, which located within liver parenchyma and were not closed to adjacent organs, such as 5^th^, 6^th^, 8^th ^and 12^th ^patient (Table 1), when using ITV_MinIP _mean MI was 0.92 ± 0.04, while using ITV_2M _it was 0.93 ± 0.03 with no significant difference between ITV_MinIP _and ITV_2M _(*p *= 0.114).

#### (3). Proportion of V_under_

**Figure 4:**
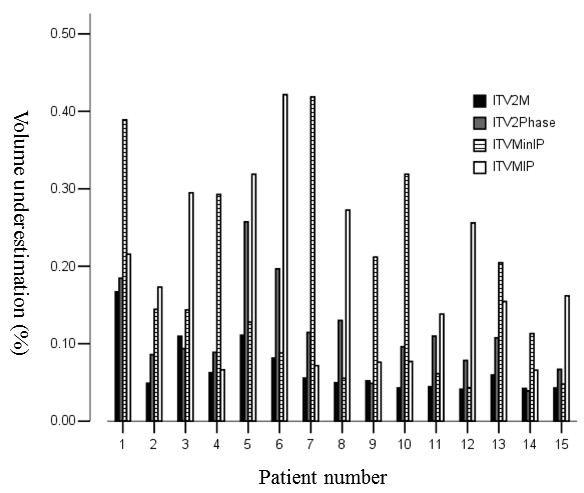
**The proportional volumetric underestimations of each ITV (ITV_2M_, ITV_2Phase_, ITV_MinIP _and ITV_MIP_) relative to the reference ITV_AllPhases _in the 15 individual patients**.

Figure 4 illustrates the proportions of V_under _in 15 patients. Compared to ITV_AllPhases_, the proportional V_under _of ITV_2M _was the lowest (0.07 ± 0.04) with the maximum of 0.17 among ITV_2Phase_, ITV_MinIP_, ITV_MIP _and ITV_2M_. While proportional V_under _of ITV_2Phase _was 0.11 ± 0.06 with the maximum of 0.26. The mean proportion of V_under _for ITV_2M _were significantly less than that for ITV_2Phase _(*p *= 0.001). However, ITV_MinIP _and ITV_MIP _underestimated larger volumes, the proportions of V_under _being 0.18 ± 0.12 and 0.18 ± 0.11, respectively.

The analyses of tumor characteristics, which would impact proportion of V_under _were as follows.

**Figure 5:**
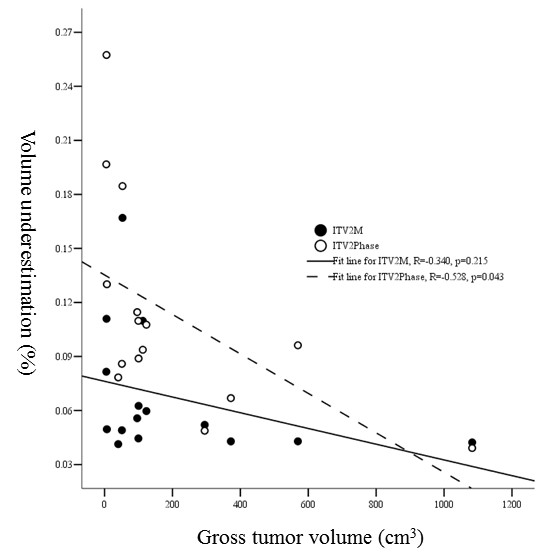
**Relationship between gross tumor volume and proportional volumetric underestimations of ITV_2M _and ITV_2Phase_**.

**1). Proportion of V_under _and tumor volume: **There were no correlations between the diameter of GTV and the proportion of V_under _for ITV_2M_, no matter the diameter over than or less than 5 cm. The proportions of V_under _were respectively 0.06 ± 0.02 and 0.08 ± 0.05 (*p *= 0.244). However, there was significant difference in proportion of V_under _between tumor size of ≤ 5 cm and > 5 cm with proportions of ITV_2Phase _being 0.08 ± 0.03 and 0.15 ± 0.07 (*p *= 0.018), respectively. For ITV_2M _as tumor size increased the proportions of V_under _did not change significantly with no correlations between them (R = -0. 340, *p *= 0.215). In contrast, for ITV_2Phase _there was negative correlation between GTV volume and the proportion of V_under _(R = -0.528, *p *= 0.043) (Figure 5).

**2). Proportion of V_under _and target motion three-dimensionally: **There was no strong correlation between 3 victor tumor motion of ≤ 10 mm and > 10 mm, and the proportions of V_under _were 0.06 ± 0.01 and 0.07 ± 0.04 (*p *= 0.480) for ITV_2M_, and 0.12 ± 0.04 and 0.10 ± 0.07 (*p *= 0.758) for ITV_2phase_.

**Figure 6:**
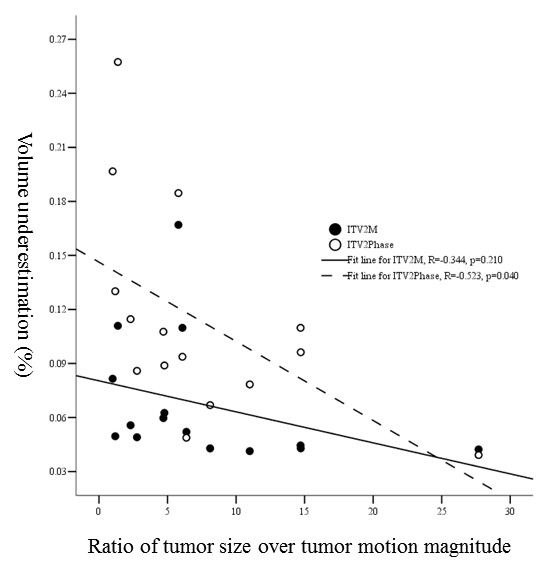
**The relationship between the ratio of tumor vertical diameter over tumor motion magnitude in cranio-caudal direction and proportional volumetric underestimations of ITV_2M _and ITV_2Phase_**.

**3). Proportion of V_under _and ratio of tumor vertical diameter over tumor motion: **For ITV_2M _there was no strong correlation between them (R = -0.344, *p *= 0.210). However, for ITV_2phase _as ratio of tumor vertical diameter over tumor motion increased the proportions of V_under _decreased significantly (R = -0.523, *p *= 0.040) (Figure 6).

**4). Proportion of V_under _and tumor density: **For both low density and mixed density tumors there was no significant difference in the underestimations, regardless ITV_2M _or ITV_2Phase_, For ITV_2M _the proportions of V_under _were 0.06 ± 0.03 and 0.07 ± 0.05 (*p *= 0.723), respectively for low density tumor and the mixed, and for ITV_2Phase _they were 0.12 ± 0.07 and 0.11 ± 0.05 (*p *= 0.680). However, when using ITV_MinIP _the underestimated proportion was 0.10 ± 0.06 for 9 low density tumors, but it was 0.29 ± 0.11 for 6 mixed density tumors (*p *= 0.001).

As for MI in 5^th^, 6^th^, 8^th ^and 12^th ^patient, when ITV_MinIP _was used the mean proportion of V_under _was 0.08 ± 0.04, while it was 0.07 ± 0.03 when ITV_2M _was used (*p *= 0.094).

#### (4). V_over_

Compared to ITV_AllPhases_, the percentages of V_over _were all less than 1% for ITV_2phase_, ITV_MinIP_, ITV_MIP _and ITV_2M_.

## Discussion

MIP method as an image post-processing is based on more complex algorithms and can be used for generating three-dimensional vascular reconstructions [14,15]. In lung cancer, MIPs are believed to be a reliable tool to generate ITVs from 4D CT data sets [10], however, it is mandatory to modify each individual MIP to improve ITV delineation for tumors adjacent to the thoracic wall, mediastinum, heart, or diaphragm [11,12]. In the current study, we also had to verify ITVs contoured on MIP and MinIP CT by overlaying it on a movie loop displaying 4D CT data and then editing it, especially for those closed to adjacent organs. Mancosu had recently proposed a semiautomatic technique, which allowed for inclusion of the residual part of ITV covered by liver and spleen cupola when using MIP algorithm. It was validated on phantom and selected patients, which revealed this possibility when lesion located near liver and spleen cupola by performing only the contours on MIP series [16]. Thus, the dedicated software needs to be developed to exclude diaphragm and chest wall in some breathing phases using 4D CT for better tumor MIP/MinIP images.

Theoretically, for tumors with homogeneous hyperdensity or hypodensity compared to the surrounding normal liver, MIP or MinIP projections should accordingly reflect the tumor trajectory across all time-resolved data sets. In patients when the lesions were homogenous low CT density, located intrahepatic, not adjacent to perihepatic organs and also small size (5^th^, 6^th^, 8^th ^and 12^th ^patient), using MinIP was also an appropriate tool with good MI of 0.92 ± 0.04 and low proportion of V_under _of 0.08 ± 0.04. Thus, ITV_MinIP _method can be used for fast contouring of liver tumor with homogeneous low CT density including respiratory motion.

However, a number of liver cancers present inhomogeneous density, results from this study showed combined MIP and MinIP fit excellently in this situation. In current study we compared MIs and proportions of V_under _resulting from ITV_2Phase_, ITV_MinIP_, ITV_MIP _and ITV_2M _contoured by 4 approaches, and found that the closest to ITV_AllPhases _was the combined one (ITV_2M_) of ITV_MinIP _and ITV_MIP_, which were contoured on MinIP and MIP series of 4D CT set because it resulted in the highest MI and lowest proportion of V_under_. Moreover, the size of tumor and the ratio of tumor vertical diameter over cranio-caudal movement did not have influence on MI or proportion of V_under _when using ITV_2M_. In other words, no matter how big the tumor was, and the tumor vertical diameter over cranio-caudal movement was small or big, the tool of ITV_2M _could always result in the best outcome.

For moving target it was also a practice to sum 2 GTVs to generate ITV, one contoured on CT image set acquired after end-inhale and holding breath and the other contoured on CT acquired after end-exhale and holding breath when 4D CT was not available. The similar method was also used for 4D CT, i.e., to contour only two extreme phases at end-inhalation and end-exhalation. However, possible hysteresis effects would be neglected as occurred in lung cancer [17,18]. Seppenwoolde and colleagues [18] reported that when the tumor was small and had a large range of motion, the separation between the positions of the images of inspiration and expiration phases was relatively obvious and the information of the intermediate breathing might not be comprehensive. Besides, the combined images of the two time phases might omit the lag of the tumor. The phenomenon was caused by the time difference among the document recorded by the computer, the transition of respiratory cycle and the transition between inspiration and expiration in a respiratory cycle. Xi [9] reported the feasibility of using limited 4D CT images for treatment planning for liver radiotherapy. As recognized by the authors, deriving ITV by two extreme phases was reasonably safe only for low and medium tumor motion amplitude (< 1.6 cm). The tumor motion in cranio-caudal direction between Xi's study and ours' were comparable, but the tumor size of our data (152.20 cm^3 ^± 242.85 cm^3^) was more diverse than Xi's (70.36 cm^3 ^± 66.23 cm^3^). Xi did not investigate the influence of tumor size on ITV determination using 4D CT data. Our study did find a smaller volume of ITV_2Phase _(186.8 cm^3^) than that of ITV_AllPhases _(200.8 cm^3^) with significant difference (*p *= 0.004). And also we found that MI and proportion of V_under _were influenced by the tumor volume and the ratio of tumor size over tumor motion magnitude significantly. Whereas, ITV_2M _was the closest to ITV_AllPhases_, and MI and proportion of V_under _were not influenced by tumor volume and the ratio of tumor size over tumor motion magnitude when using tool of ITV_2M_.

## Conclusion

To reduce the workload of contouring multiple GTVs in 4D CT data sets, contouring only two extreme phases is appropriate only when tumor volume is big and tumor motion magnitude is relatively small. For hepatic malignancies with inhomogeneous density we found that the method of using ITV_2M _was a more reliable and appropriate tool for generating ITVs from 4D CT data sets, compared to the others.

## Competing interests

The authors declare that they have no competing interests.

## Authors' contributions

ZJD and JGL designed the study. LJ, WJZ and ZJD did the study and wrote the manuscript. JGL was responsible for manuscript revision and submission. WJZ and XZY were involved in 4D CT simulation and data analysis. All authors read and approved the final version of the manuscript.
